# Effects of sub-lethal teratogen exposure during larval development on egg laying and egg quality in adult *Caenorhabditis elegans*

**DOI:** 10.12688/f1000research.8934.1

**Published:** 2016-12-28

**Authors:** Alexis Killeen, Caralina Marin de Evsikova

**Affiliations:** 1 Department of Molecular Medicine, Morsani College of Medicine, University of South Florida, Tampa, USA

**Keywords:** Arsenic, Benzo-α-pyrene, Biocides, Bisphenol A, Cadmium, Cigarette smoke, Combustion Pollutants, Diethylstilbestrol, Egg Laying, Egg Hatching, Egg Viability, Endocrine Disruptors, Fenthion, Nicotine, Tributyltin, Triclosan

## Abstract

*Background: *Acute high dose exposure to teratogenic chemicals alters the proper development of an embryo leading to infertility, impaired fecundity, and few viable offspring. However, chronic exposure to sub-toxic doses of teratogens during early development may also have long-term impacts on egg quality and embryo viability. *Methods: *To test the hypothesis that low dose exposure during early development can impact long-term reproductive health, *Caenorhabditis elegans* larvae were exposed to 10 teratogens during larval development, and subsequently were examined for the pattern of egg-laying and egg quality (hatched larvae and embryo viability) as gravid adults. **After the exposure, adult gravid worms were transferred to untreated plates and the numbers of eggs laid were recorded every 3 hours, and the day following exposure the numbers of hatched larvae were counted. *Re* *sults: *While fecundity and fertility were typically impaired by teratogens, unexpectedly, many teratogens initially increased egg-laying at the earliest interval compared to control but not at later intervals. However, egg quality, as assessed by embryo viability, remained the same because many of the eggs (<50%) did not hatch. *Conclusions: *Chronic, low dose exposures to teratogens during early larval development have subtle, long-term effects on egg laying and egg quality.

## Introduction

Teratogens are agents that negatively impact reproduction and embryonic development and include radiation, maternal infections, pharmaceuticals, and chemicals ( Wilson, 1973). Numerous chemicals act as teratogens that adversely affect human health, with the time period of exposure as a critical factor determining teratogen susceptibility. For instance, maternal and prenatal teratogen exposure is associated with birth defects, spontaneous abortion, and stillbirth, and sometimes cancer in the reproductive tract of progeny (Reed *et al.,* 2013; Sanders *et al*., 2014; Wigle *et al*., 2008). Previous studies demonstrated that acute, high-dose teratogen exposure causes reproductive decline, but the long-term ramifications of low dose teratogen exposure during early development later in life remain unknown ( Allard & Colaiácovo, 2010; Parodi *et al*., 2015). In this study, we used egg-laying, hatching, and offspring viability assays phenotyping screen throughout early development after exposing *Caenorhabditis elegans* to sub-lethal doses from in three classes of teratogens, including biocides, endocrine disruptors, and combustion pollutants, to detect impacts on reproductive phenotype in adults.

In addition, identification of the time frame, yielding the maximal egg-laying, after teratogen exposure, is critical for behavioral and developmental experiments requiring an aged-matched offspring population. The results of our study guide experimental procedures to obtain the requisite population size for subsequent experiments employing 3 hours egg-laying time window to achieve developmentally synchronous, aged-matched offspring population for subsequent experiments assessing long-term effects.

## Methods

### *Caenorhabditis elegans* maintenance

N2 ancestral Bristol strain of *C. elegans* (CGC, Minneapolis, MN, USA) were maintained on NGM lite plates (N1005, US Biologicals, Salem, MA, USA) with bacterial lawns at 25±0.5°C for all experiments. Bacterial lawns were grown on Petri dishes (10, 60, 100 mm Corning, USA) overnight using 10, 20 or 60 µl of 1X *E. coli* OP50 (CGC, Minneapolis, MN, USA; 1X=OD _600_=8.0×10 ^8^ cells/mL).

### Chemicals

Teratogens, except for cigarette smoke extract, were purchased from Sigma Aldrich (St. Louis, MO, USA): tributyltin-chloride (0.1 µM, T50202), cadmium-chloride (0.5 µM, C2554), benzo-α-pyrene (0.5 µM, B1760), nicotine (0.5 µM, N-3876), bisphenol-A (10 µM, 239658), diethylstilbestrol (10 µM, D4628), arsenic(III) oxide (0.5 µM, A1010), triclosan (0.1 µM PHR1338), fenthion (0.1, 1 µM, 36552). Cigarette smoke extract (0.1 µM) was purchased from Murty Pharmaceuticals (0.1 µM, Lexington, KY, USA). Stock solutions of teratogens were made in DMSO or water, and final concentrations were adjusted to contain 0.01% DMSO (ACS grade, Fisher Scientific, USA). Sub-lethal doses were chosen based on preliminary studies (unpublished McIntyre, Killeen & Marín de Evsikova).

### Experimental procedure

**Figure 1.**
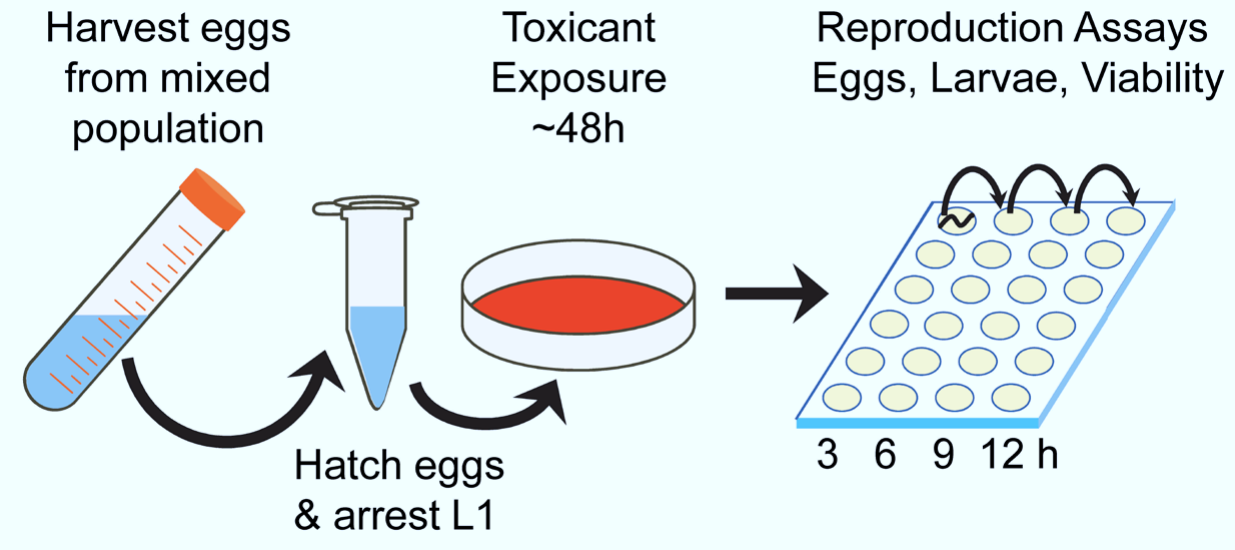
Study experimental design and procedure. An age-synchronized population of *C. elegans* L1 larvae was exposed to 10 known teratogens at sub-lethal, micromolar concentrations for 48 hours. Gravid adult worms were transferred to pure NGM with bacterial lawns and the number of eggs laid was recorded every 3 hours up to 12 hours. Hatched larvae counted the following day.

The experimental design for all studies is depicted in Figure 1. A mixed population of worms was used to prepare a developmentally synchronized population at L1 larval stage ( Stiernagle, 2006). All plates were coded to prevent experimenter bias. Larvae were transferred onto vehicle or teratogen exposure plates (with bacterial lawns) and cultivated for 48 hours at 25°C ( Figure 1). Adult worms from each group were washed 3 times in M9 buffer and transferred into a well in the first row of a 24-well NGM plate. These worms were moved to an adjacent well every three hours for 12 hours. Laid eggs, and the following day, hatched embryos were counted. Embryo viability was determined as the ratio of hatched larvae to number of eggs. Experiments were repeated six times (n=60 worms/group, yielding N=660 worms), albeit fenthion, which was repeated twice at 1 µM (n=20), and after unexpectedly curtailed egg-laying, the dose was decreased to 0.1 µM (n=40). Statistically significant differences were determined after outlier analysis using SPSS v. 23 software (IBM, USA) by ANOVA followed by post hoc or nonparametric (Χ ^2^ or Fisher’s Exact Tests) for fecundity, fertility, eggs laid, hatched larvae, and embryo viability among all groups with significance set at p<0.05.

## Results

**Figure 2.**
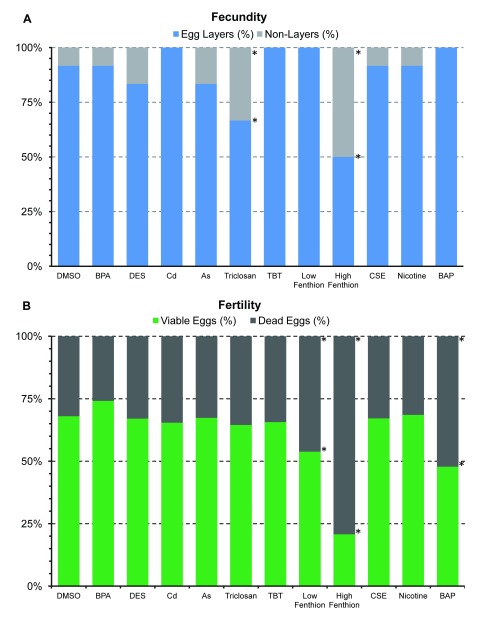
Effects of teratogens on fecundity ( **A**) and fertility ( **B**). The overall percentage of worms that laid eggs shown as fecundity and the overall percentage of viable and dead eggs as a measure of fertility. Asterisks indicate *P*<0.05.

**Figure 3.**
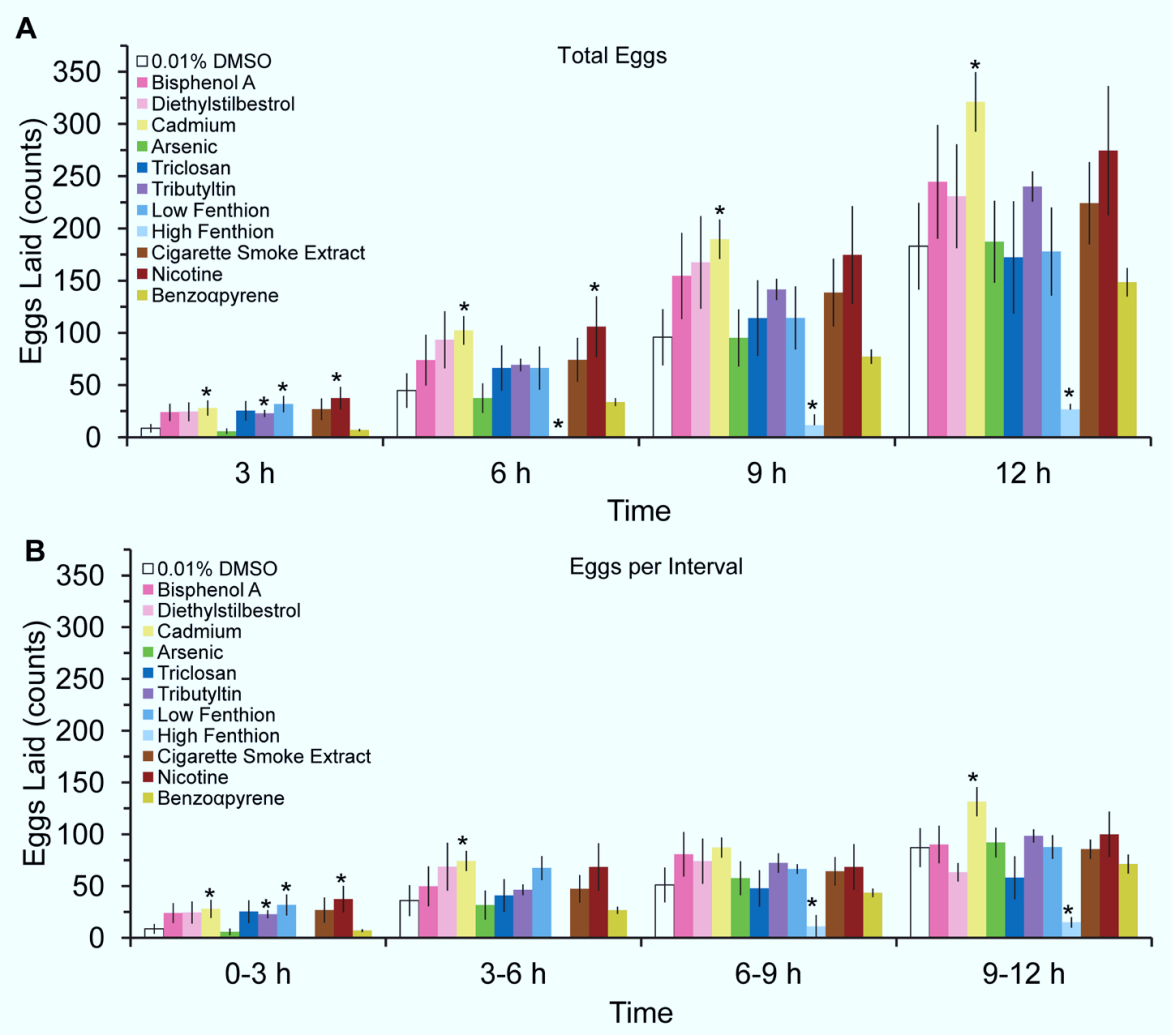
Effects of teratogens on egg-laying. Temporal pattern of ( **A**) cumulative eggs laid at 3, 6, 9 and 12 hours post-exposure, and ( **B**) rate of eggs laying in 3 hours intervals (mean ± sem). Asterisks indicate *P*<0.05.

**Figure 4.**
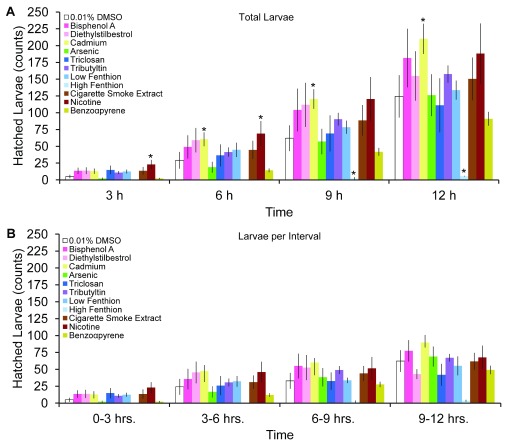
Effects of teratogens on hatched larvae. Temporal pattern of ( **A**) cumulative hatched larvae from eggs laid at 3, 6, 9 and 12 hours post-exposure to teratogens and ( **B**) number of hatched larvae per teratogen group or control in 3 hours intervals (mean ± sem). Asterisks indicate *P*<0.05.

**Figure 5.**
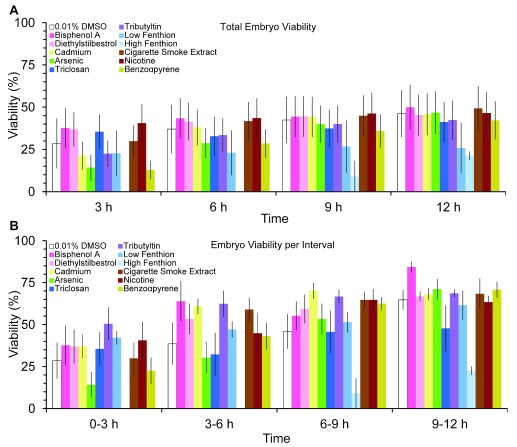
Effects of teratogens on embryo viability. Temporal pattern of ( **A**) overall embryo viability at 3, 6, 9 and 12 hours post-exposure to teratogens and ( **B**) embryo viability at 3 hours intervals. Embryo viability calculated as the ratio of hatched larvae/eggs laid X 100% (mean ± sem). Asterisks indicate *P*< 0.05.

Fecundity and fertility, measures of reproductive fitness of the organism, were decreased after worms were exposed to the biocides, triclosan and fenthion, and combustion pollutant, benzo-α-pyrene (BAP; Χ ^2^= 11.27, 5.21, and 83.1, p<0.05, respectively, Figure 2A & B). Despite the overall detrimental effect on fecundity and fertility, the initial temporal pattern of egg laying was increased by some teratogens. The cumulative amount of eggs laid, irrespective of teratogen or vehicle, increased over time except for 1 µM dose of fenthion ( Figure 3A, *F*(3,162)=119, p<0.05). Furthermore, chronic larval exposure to some low dose teratogens, such as nicotine, cadmium, and tributyltin, increased egg laying and cumulatively produced more eggs than vehicle control ( Figure 3A). Unexpectedly, many teratogens, except for arsenic and benzo-α-pyrene, produced more eggs during the 0–3 hours interval compared to vehicle ( Figure 3B), although this interval had the overall lowest yield of eggs. While the greatest amount of egg-laying occurred during the 9–12 hours compared to the 0–3 hours interval, no differences in the amount of eggs laid were detected among the intervals at 3–6 hours, 6–9 hours, and 9–12 hours (p>0.05). Hatched larvae, unlike eggs, did not increase at any interval after teratogen exposure ( Figure 4B, *F*(33,162)=1.051) but total hatched larvae increased cumulatively by 12 hours ( *F*(3,162)=24.8, p<0.05). Despite these increases, egg quality did not improve after teratogen exposure as overall embryo viability was similar across groups and time points ( Figure 5A *F*(33,162) = 1.015, *P*>.4) with lowest viability at the first interval ( Figure 5B) albeit not significant.

Dataset of screening sub-lethal teratogens on egg laying, hatching and viability in adult C. elegans during larval development Data files contain raw data of the effect of teratogens on larva hatching, egg laying and viability in *C. elegans*. ( Killeen & Marín de Evsikova, 2016) Click here for additional data file. Copyright: © 2016 Killeen A and Marin de Evsikova C 2016 Data associated with the article are available under the terms of the Creative Commons Zero "No rights reserved" data waiver (CC0 1.0 Public domain dedication).

## Discussion

Early larval exposure to low dose teratogens alters egg-laying without improving embryo viability at later time points, which result in some overall detrimental impacts on fecundity and fertility. An improvement in embryonic viability at the latter time intervals was expected because adult hermaphrodites replenish their entire gonad every 6.5 hours ( Hirsh *et al*., 1976), which implies that eggs laid during or after the 6–9 hour interval hours would have not been exposed to teratogens and egg-laying and egg quality should have increased at the latter two intervals compared to earlier intervals of 0–3 hours and 3–6 hours. While egg-laying improved during at the 9–12 hours interval, egg quality did not improve compared to either 0–3 or 3–6 hours intervals, as measured by embryo viability. Due to specifics of gametogenesis in *C. elegans*, it is possible that teratogens may affect egg quality by altering sperm cell quality ( Hirsh *et al*., 1976). It is also possible that chronic exposure to teratogens may be exacerbated by their extended bioactivity, or in some cases, through actions of their metabolites. These results indicate greater egg harvests are necessary to obtain an offspring population to further assess long-term teratogen effects on offspring.

This procedure using *C. elegans* not only identifies sub-lethal teratogen concentrations with potential long-term effects upon egg laying and quality, it provides the experimental platform to expand knowledge to prevent birth defects by developing interventions to ameliorate chronic subtoxic, teratogenic exposures upon the embryo, and is an initial step towards designing and testing safe therapeutics to be used before and during gestation. Developing phenotyping screens with simple organisms, such as *C. elegans*, is an efficient way to identify putative teratogens with potential long-term effects to further knowledge on the underlying developmental processes susceptible to environmental insults, and reveals the basis of how environment affects and shapes development.

## Data availability

The data referenced by this article are under copyright with the following copyright statement: Copyright: © 2016 Killeen A and Marin de Evsikova C

Data associated with the article are available under the terms of the Creative Commons Zero "No rights reserved" data waiver (CC0 1.0 Public domain dedication). 

F1000Research: Dataset1. Dataset of screening sub-lethal teratogens on egg laying, hatching and viability in adult *C. elegans* during larval development, 10.5256/f1000research.8934.d124253 ( Killeen & Marín de Evsikova, 2016).

## References

[ref-1] Allard P Colaiácovo MP: Bisphenol A impairs the double-strand break repair machinery in the germline and causes chromosome abnormalities. *Proc Natl Acad Sci U S A.* 2010;107(47):20405–20410. 10.1073/pnas.1010386107 21059909 PMC2996676

[ref-2] Hirsh D Oppenheim D Klass M: Development of the reproductive system of *Caenorhabditis elegans*. *Dev Biol.* 1976;49(1):200–219. 10.1016/0012-1606(76)90267-0 943344

[ref-3] Killeen A Marín de Evsikova C: Dataset 1 in: Screening sub-lethal teratogens during larval development for long-term effects on egg laying, hatching, and viability in adult *Caenorhabditis elegans*. *F1000Research.* 2016 Data Source 10.12688/f1000research.8934.1 PMC5247790 28163903

[ref-4] Parodi DA Sjarif J Chen Y: Reproductive toxicity and meiotic dysfunction following exposure to the pesticides Maneb, Diazinon and Fenarimol. *Toxicol Res (Camb).* 2015;4(3):645–654. 10.1039/C4TX00141A 25984295 PMC4433152

[ref-5] Reed CE Fenton SE: Exposure to Diethylstilbestrol during Sensitive Life Stages: A legacy of Heritable Health Effects. *Birth Defects Res C Embryo Today.* 2013;99(2):134–146. 10.1002/bdrc.21035 23897597 PMC3817964

[ref-6] Sanders AP Desrosiers TA Warren JL: Association between arsenic, cadmium, manganese, and lead levels in private wells and birth defects prevalence in North Carolina: a semi-ecologic study. *BMC Public Health.* 2014;14(14):955. 10.1186/1471-2458-14-955 25224535 PMC4190372

[ref-7] Stiernagle T: Maintenance of *C.elegans.* *WormBook.*ed. The *C.elegans*Research Community, WormBook,2006;1–11. 10.1895/wormbook.1.101.1 18050451 PMC4781397

[ref-8] Wigle DT Arbuckle TE Turner MC: Epidemiologic Evidence of Relationships Between Reproductive and Child Health Outcomes and Environmental Chemical Contaminants. *J Toxicol Environ Health B Crit Rev.* 2008;11(5–6):373–517. 10.1080/10937400801921320 18470797

[ref-9] Wilson JG: Environment and Birth Defects (Environmental Science Series). London: Academic Press;1973.

