# Supplementary material for: Effects of sub-lethal teratogen exposure during larval development on egg laying and egg quality in adult Caenorhabditis elegans
Source: F1000Res. 2016 Dec 28;5:2925. [Version 1] doi: 10.12688/f1000research.8934.1 (PMC5247790; doi:10.12688/f1000research.8934.1)
Supplement: Peer review history

# Peer review: Referee response for version 1

**DOI:** 10.5256/f1000research.9613.r18823

**Published:** 2017-01-16

**Authors:** Keith P. Choe

**Affiliations:** 1 Department of Biology, Genetics Institute, University of Florida, Gainesville, FL, USA

This is a study providing proof of principle for an approach to screening potential teratogens using the nematode model C. elegans.  The small size, simple culturing methods, and genetic tractability of C. elegans make it useful for rapid screening of compounds for biological activity and for identifying genetic factors that contribute to compound mode of action.

 The results demonstrate the ability of the assay to detect compound effects on egg production and embryo viability.  The authors might comment of embryo viability; the percent of eggs hatching is pretty low (generally under 50% even with vehicle control).  Embryo viability on agar plates is usually far higher.

 A useful modification of the assay would be to extend monitoring for the whole egg-laying period of 3-4 days to measure total eggs laid and to measure the latency of recovery on egg production and embryo viability.

I have read this submission. I believe that I have an appropriate level of expertise to confirm that it is of an acceptable scientific standard.

# Peer review: Referee response for version 1

**DOI:** 10.5256/f1000research.9613.r19052

**Published:** 2017-01-06

**Authors:** Keith T. Jones

**Affiliations:** 1 Centre for Biological Sciences, Faculty of Natural and Environmental Sciences, University of Southampton, Southampton, UK

This is a well conducted experiment examining the influence of various teratogens on the egg laying rate and embryo quality, and progeny, in C. elegans. There is an expected fall off in the fertility of worms exposed, however the actual rate of egg laying on initial exposure was raised.

 My only comment would be that egg laying in worms is associated with/ needs worm movement. Thus you can get a ‘bag of worms’ phenotype if all movement is blocked. Hypothetically worm irritancy by noxious exposure in itself may promote movement, which then raises egg laying rate. It may be worthwhile commenting on this as a possibly explanation.

I have read this submission. I believe that I have an appropriate level of expertise to confirm that it is of an acceptable scientific standard.
